# Two decades of mortality trends in pancreatic cancer and diabetes mellitus: A retrospective cross-sectional study of the United States population (1999–2020)

**DOI:** 10.1097/MD.0000000000046372

**Published:** 2026-05-12

**Authors:** Asad Ali Ahmed Cheema, Aqsa Shaikh, Neelam Kumari, Muhammad Abdul Rafay, Abuzar Khan

**Affiliations:** a International School of Medicine, International University of Kyrgyzstan, Bishkek, Kyrgyzstan; b Department of Medicine, Liaquat National Hospital and Medical College, Karachi, Pakistan; c Department of Medicine, WellSpan York Hospital, York, PA; d Department of Medicine, Shaheed Zulfiqar Ali Bhutto Medical University, Islamabad, Pakistan; e Department of Medicine, Khyber Medical College, Peshawar, Pakistan.

**Keywords:** CDC WONDER, diabetes mellitus, mortality, pancreatic cancer, US

## Abstract

The incidence of pancreatic cancer (PC) is on the rise, making it a leading cause of cancer deaths, particularly with aging, obesity, and diabetes mellitus (DM). However, trends in mortality among individuals affected by both conditions in the United Stated (US) remain inadequately understood. This study aims to examine national mortality trends from 1999 to 2020 among adults aged ≥45 years with coexisting PC and DM. We investigated death trends related to PC and DM in adults aged 45 years and older between 1999 and 2020 using mortality data from the CDC Wide-Ranging Online Data for Epidemiologic Research (CDC WONDER) system. We calculated age-adjusted mortality rates (AAMRs) and crude mortality rates (CMRs) per 10,00,000 people. The annual percent change (APC) and associated 95% confidence intervals (CIs) were used to assess temporal patterns. We stratified the study by sex, race/ethnicity, year, urban/rural status, and US region to identify demographic and geographic inequalities. We performed a sensitivity analysis restricting deaths to PC as underlying cause with DM contributory, recalculating AAMRs/average annual percent changes using identical Joinpoint settings. From 1999 to 2020, 60,213 deaths were attributed to the combined effects of PC and DM among adults aged ≥45 years. The AAMR was higher in males (28.68 per 10,00,000) than in females (18.91 per 10,00,000). The non-Hispanic (NH) Black population had the highest AAMR (35.50 per 10,00,000), followed by Hispanic or Latino (26.57), NH White (22.09), and NH Asian or Pacific Islander (20.06). The age-specific CMR peaked in individuals ≥85 years (75.01 per 10,00,000). Regionally, the West (25.92) and Midwest (24.71) had the highest mortality, with elevated rates in rural areas (26.13). Nebraska recorded the highest state-level mortality (37.60 per 10,00,000). Sensitivity results confirmed increases overall (average annual percent change: 1.50; 95% CI: 0.98–2.02), steeper in males (1.99) and rural residents (2.33) versus females (0.94) and urban (1.24). From 1999 to 2020, AAMR due to coexisting PC and DM rose significantly across US demographic and geographic groups. These findings highlight the growing dual burden of these conditions and the urgent need for targeted prevention and equitable access to care.

**Key Points**

- Pancreatic cancer-related mortality with coexisting diabetes has risen significantly among US adults aged ≥45 years from 1999 to 2020.
- Males and non-Hispanic Black individuals experienced the highest mortality burden.
- Marked geographic disparities were observed, with rural regions and several states showing disproportionately high mortality.
- Sensitivity analysis confirmed consistent upward trends, particularly among males and rural residents, demonstrating the robustness of the observed mortality increases.
- These findings underscore the urgent need for targeted prevention, early detection, and equitable access to care for high-risk populations.

## 1. Introduction

Pancreatic cancer (PC) is the third-deadliest cancer, with a 5-year survival rate of 12.8% reducing to 3.1% in the case of distant metastasis.^[1]^ According to the trends, the age-adjusted mortality rates (AAMR) for new PC cases have been rising by an average of 0.2% every year over the last decade. They are estimated to be the second primary contributor to cancer deaths by 2030.^[1,2]^ In parallel, diabetes mellitus (DM), a known risk factor for PC, ranks as the eighth leading global cause of disease burden and is expected to rise to second place by 2050.^[3]^

PC and DM share a complex, bidirectional relationship. In addition to being a risk factor for PC, new-onset DM in the older population has been frequently reported in the literature recently as an alarming indication of PC development.^[4]^ The upward trends of PC are attributed to an aging population, sociodemographic index, and increased prevalence of DM.^[5]^ This upsurge in the burden of disease is expected to result in a significant increase in both economic and healthcare costs.^[6]^ However, data have been scarce on overall trends of mortality related to PC in association with DM to suggest the actual public health burden thus far in the setting of disproportionately lacking screening and treatment modalities.

Previous research has primarily investigated PC and DM as separate entities, without examining their combined impact on mortality.^[7,8]^ Despite the recognized biological and clinical connection between these diseases, there remains a lack of population-based evidence evaluating national mortality trends in individuals with both conditions.

This study is the first to utilize nationally representative Center for Disease Control and Prevention Wide-Ranging Online Data for Epidemiologic Research (CDC WONDER) data to comprehensively assess mortality patterns from 1999 to 2020 in United States (U.S) adults aged 45 years and older living with coexisting PC and DM. Exploring demographic, racial, geographic, and urban–rural variations, it provides novel insights into the joint mortality burden of these conditions. It underscores the urgent need for targeted prevention, early detection, and policy initiatives to address this growing public health concern.

## 2. Materials and methods

### 2.1. Study setting and population reference

Mortality information was extracted from the CDC WONDER database. Adults aged 45 and above were assessed for mortality due to both PC and DM in the US from 1999 to 2020. To identify deaths attributed to both PC and DM, the International Statistical Classification of Diseases and Related Health Problems-10th Revision (ICD-10) codes were used. PC was identified using codes C25.0 to C25.3 (malignant neoplasms of the head, body, tail, and duct of the pancreas), C25.4 (endocrine pancreas), and C25.7 to C25.9 (other specified sites, overlapping lesions, and unspecified parts of the pancreas). DM was identified using codes E10 through E14, encompassing all major subtypes of DM.^[9,10]^ Only cases in which both PC and DM were listed as contributing causes of death on the death certificate, as recorded in the multiple cause of death files, were included.

### 2.2. Data abstraction

Abstracted data included year, population size, demographics, place of death, urban/rural status, states, and regions. The following variables were included as demographics: gender, age, race, and location of death, including medical facilities (outpatient, emergency room, inpatient, and death on arrival or status unknown), home, hospice, and long-term care facility or nursing home. Ethnicity or race was categorized as follows: Hispanic or Latino, non-Hispanic (NH) White, NH Black or African American, NH Asian or Pacific Islander, and NH American or Alaska Native. This information is derived from death certificates and has been utilized in earlier research projects that employed the CDC WONDER database.^[11]^ The population was categorized into urban and rural areas according to the National Centre for Health Statistics’ Urban–Rural Classification Scheme. Urban classification included large metropolitan regions with populations over 1 million and small to medium metropolitan regions with populations between 50,000 and 1 million. In contrast, rural areas were classified as regions with populations of fewer than 50,000, according to the 2013 US Census classification.^[11]^ For regional analysis, the US was divided into 4 regions Northeast, Midwest, South, and West according to the classification provided by the US Census Bureau.^[12]^

### 2.3. Statistical analysis

We calculated AAMRs and crude rates per 10,00,000 people from 1999 to 2020, categorizing by year, gender, ethnicity, racial background, state, and urban–rural classification, with 95% confidence intervals (CIs). The crude mortality rate (CMR) was calculated by dividing the total deaths due to PC and DM by the US population of the respective year. To assess changes in mortality trends related to PC and DM, we estimated the percent change in AAMRs per 10,00,000 people between 1999 and 2020, utilizing the 2000 US population as a standard reference.^[13]^ AAMRs were used for all analyses, except for age-specific categories, where the CMR was used instead. National yearly trends in PC and DM-related mortality were *assessed* using the Joinpoint Regression Program (Version 5.3.0, National Cancer Institute, Bethesda) by computing the annual percent change (APC) in AAMRs, along with 95% CIs. This technique utilized log-regression models to detect significant changes in AAMR over time, in the presence of temporal variability. APCs were deemed as rising or decreasing if the slope revealing differences in mortality deviated significantly from 0, as determined by 2-tailed *t*-testing. A *P*-value of <.05 was considered statistically significant.

To account for potential misattribution of the cause of death and to test the robustness of our findings, a sensitivity analysis was conducted, restricting the dataset to cases where PC was recorded as the underlying cause of death and DM as a contributing cause. AAMR and average annual percent changes (AAPCs) were recalculated for this subset using identical statistical methods and Joinpoint regression parameters. The results of this sensitivity analysis are presented separately in the results section. All rates are population-based (per 10,00,000 US residents) and were age-adjusted to the 2000 US standard population. Analyses did not adjust for secular changes in DM prevalence; therefore, trends reflect the population-level mortality burden of coexisting PC and DM rather than mortality conditional on having DM. As such, increases in AAMR may reflect more individuals living with DM, changes in case-fatality, population aging, or coding/ascertainment differences. Results should be interpreted with this scope in mind.

### 2.4. Protocol approval and patient consent

Since the study adhered to Strengthening the Reporting of Observational Studies in Epidemiology (STROBE) principles and used de-identified, publicly available data from CDC WONDER, Institutional Review Board (IRB) approval was not necessary. Any information that CDC WONDER deemed untrustworthy or suppressed was not included.^[14]^

## 3. Results

A total of 60,213 individuals aged 45 and over died due to PC and DM from 1999 to 2020. The AAMR showed a significant upward trend from 19.72 per 10,00,000 in 1999 to 28.61 per 10,00,000 in 2020 (Fig. S1 and Table S1, Supplemental Digital Content, https://links.lww.com/MD/Q856). Among total deaths, location data was recorded for 59,472, revealing that 46.46% took place at home, 25.13% were reported in the healthcare setting, 15.36% in nursing homes or long-term care facilities, and 7.86% in hospices. For 0.15% fatalities, place of death was unknown, 0.05% were declared dead on arrival in a medical facility, and 4.98% were in other places (Table S2, Supplemental Digital Content, https://links.lww.com/MD/Q856).

### 3.1. Yearly mortality trends for PC and DM

**Figure 1.**
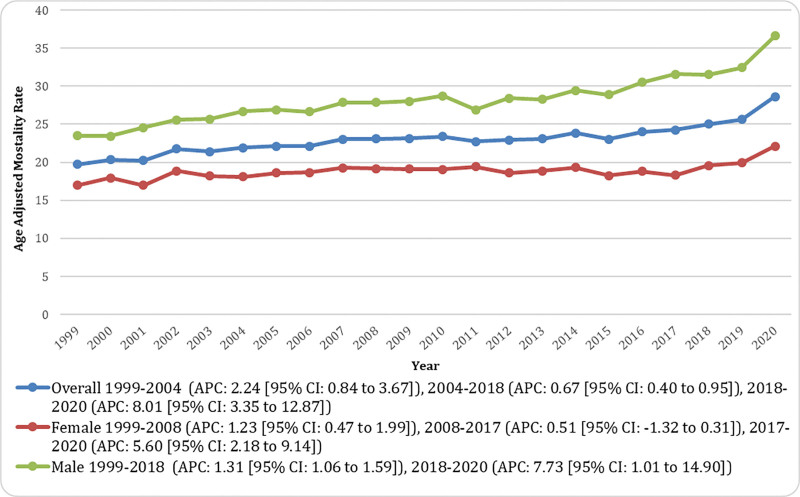
Overall and sex-stratified pancreatic cancer and diabetes mellitus-related age-adjusted mortality rates (AAMRs) per 10,00,000 in adults in the United States, 1999 to 2020. Trends in AAMRs are presented for males, females, and overall, with Joinpoint-derived annual percent change (APC) and 95% confidence intervals shown for each trend segment. AAMR = age-adjusted mortality rate, APC = annual percent change, CI = confidence interval.

The AAMR increased from 19.72 per 10,00,000 in 1999 to 28.61 per 10,00,000 in 2020. From 1999 to 2004, the AAMR rose significantly (APC: 2.24 [95% CI: 0.84–3.67]). Between 2004 and 2018, the increase continued but at a slower yet significant pace (APC: 0.67 [95% CI: 0.40–0.95]). A sharp surge was observed from 2018 to 2020 (APC: 8.01 [95% CI: 3.35–12.87]). Across the entire study period, the overall trend showed a significant increase (AAPC: 1.72 [95% CI: 1.20–2.25]; Fig. 1; Tables S3 and S4, Supplemental Digital Content, https://links.lww.com/MD/Q856).

### 3.2. Gender-wise stratification of AAMR

Across the study period, the AAMR was consistently higher in males than in females (overall AAMR: 28.68 per 10,00,000 [95% CI: 28.36–29.00] for men vs 18.91 per 10,00,000 [95% CI: 18.68–19.13] for women). Among men, the AAMR increased from 23.51 per 10,00,000 in 1999 to 31.54 per 10,00,000 in 2018 (APC: 1.31 [95% CI: 1.06–1.59]) and then rose sharply to 36.60 per 10,00,000 by 2020 (APC: 7.73 [95% CI: 1.01–14.90]). For women, the AAMR increased from 16.96 per 10,00,000 in 1999 to 19.15 per 10,00,000 in 2008 (APC: 1.23 [95% CI: 0.47–1.99]), followed by a nonsignificant decline to 18.31 per 10,00,000 in 2017 (APC: −0.51 [95% CI: −1.32–0.31]) and then a significant rise to 22.11 per 10,00,000 in 2020 (APC: 5.60 [95% CI: 2.18–9.14]). Overall, mortality rates were significantly higher among men than women throughout the study period (Fig. 1; Tables S3 and S4, Supplemental Digital Content, https://links.lww.com/MD/Q856).

### 3.3. AAMR by race and ethnicity

**Figure 2.**
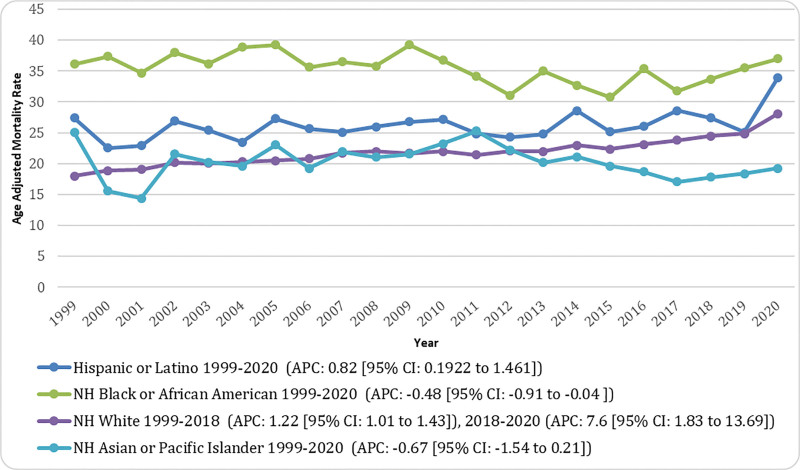
Race-stratified pancreatic cancer and diabetes mellitus-related age-adjusted mortality rates (AAMRs) per 10,00,000 in adults in the United States, 1999 to 2020. Trends are shown for Hispanic or Latino, non-Hispanic (NH) Black or African American, NH White, NH Asian or Pacific Islander, with Joinpoint-derived annual percent change (APC) and 95% confidence intervals shown for each trend segment. AAMR = age-adjusted mortality rate, APC = annual percent change, CI = confidence interval, NH = non-Hispanic.

Across racial groups, the highest AAMRs were consistently observed among NH Black or African American individuals, followed by Hispanic or Latino, NH White, and NH Asian or Pacific Islander populations. Among NH Black or African American individuals, the AAMR was 36.15 per 10,00,000 in 1999 and remained relatively stable, reaching 36.98 per 10,00,000 in 2020, showing a small but statistically significant decline (APC: −0.48 [95% CI: −0.91 to −0.04]). For NH White individuals, the AAMR increased steadily from 18.01 per 10,00,000 in 1999 to 24.49 per 10,00,000 in 2018 (significant rise, APC: 1.22 [95% CI: 1.01–1.43]), followed by a marked and significant increase to 28.11 per 10,00,000 in 2020 (APC: 7.60 [95% CI: 1.83–13.70]). Among Hispanic or Latino individuals, the AAMR rose gradually from 27.39 per 10,00,000 in 1999 to 33.92 per 10,00,000 in 2020 (significant increase, APC: 0.82 [95% CI: 0.19–1.46]). In contrast, NH Asian or Pacific Islander individuals experienced a nonsignificant decline in AAMR, decreasing from 25.03 per 10,00,000 in 1999 to 19.28 per 10,00,000 in 2020 (APC: −0.67 [95% CI: −1.54–0.21]). Data for NH American Indian or Alaska Native individuals were excluded from the Joinpoint analysis due to unreliability (Fig. 2; Tables S3 and S5, Supplemental Digital Content, https://links.lww.com/MD/Q856).

### 3.4. CMR by age group

**Figure 3.**
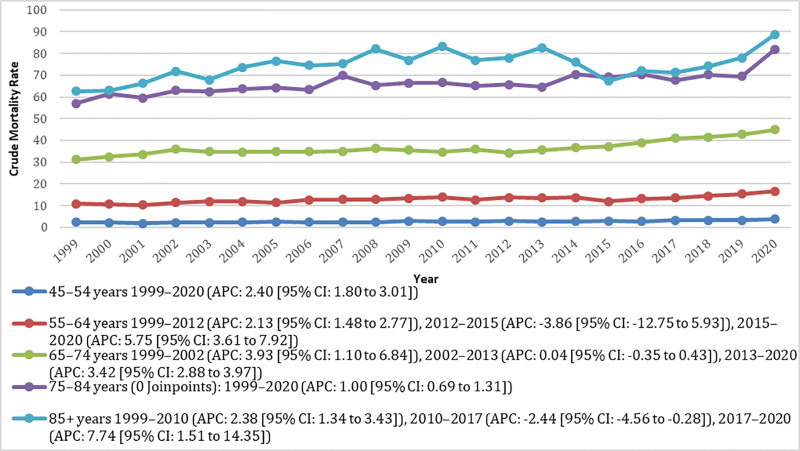
Age-stratified pancreatic cancer and diabetes mellitus-related crude mortality rates (CMRs) per 10,00,000 in adults in the United States, 1999 to 2020. Trends are shown for age categories 45 to 54, 55 to 64, 65 to 74, 75 to 84, 85+, with Joinpoint-derived annual percent change (APC) and 95% confidence intervals shown for each trend segment. APC = annual percent change, CI = confidence interval, CMR = crude mortality rate, NH = non-Hispanic.

The overall CMR per 10,00,000 population for the 45 to 54, 55 to 64, 65 to 74, 75 to 84, and ≥85-year age groups were 2.63 (95% CI: 2.52–2.73), 13.05 (95% CI: 12.80–13.31), 37.08 (95% CI: 36.55–37.61), 66.60 (95% CI: 65.67–67.52), and 75.01 (95% CI: 73.46–76.56), respectively. In the 45 to 54-year group, mortality increased steadily from 1999 to 2020 (APC: 2.40 [95% CI: 1.80–3.00]). For the 55 to 64-year group, mortality rose significantly from 1999 to 2012 (APC: 2.12 [95% CI: 1.48–2.77]), declined nonsignificantly from 2012 to 2015 (APC: −3.86 [95% CI: −12.75 to 5.92]), and then increased again through 2020 (APC: 5.75 [95% CI: 3.61–7.92]). The 65 to 74-year group showed a significant rise between 1999 and 2002 (APC: 3.92 [95% CI: 1.10–6.84]), followed by a nonsignificant change from 2002 to 2013 (APC: 0.04 [95% CI: −0.35 to 0.43]) and then a significant increase through 2020 (APC: 3.42 [95% CI: 2.88–3.97]). The 75 to 84-year-old group demonstrated a consistent upward trend across the study period (APC: 1.00 [95% CI: 0.69–1.31]). Among individuals aged ≥85 years, mortality increased significantly from 1999 to 2010 (APC: 2.38 [95% CI: 1.34–3.42]), then declined significantly through 2017 (APC: −2.43 [95% CI: −4.56 to −0.28]), followed by a sharp increase to 2020 (APC: 7.73 [95% CI: 1.51–14.35]; Fig. 3; Table S6, Supplemental Digital Content, https://links.lww.com/MD/Q856).

### 3.5. Stratified by geographical region

#### 3.5.1. State

**Figure 4.**
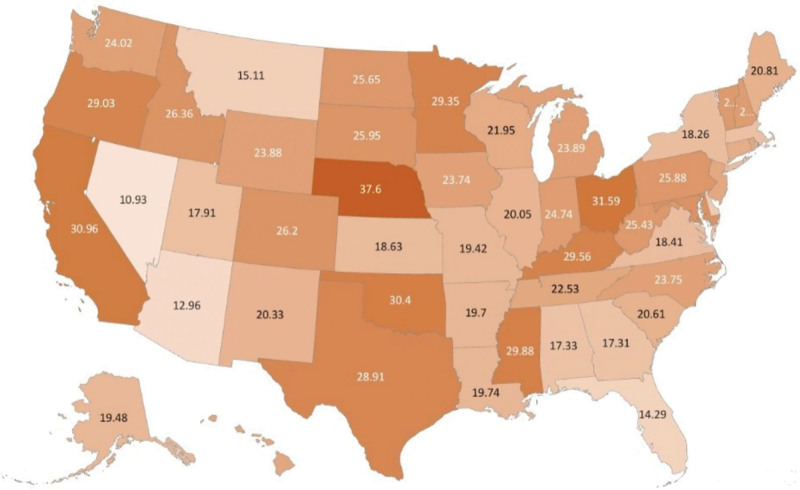
State-stratified pancreatic cancer and diabetes mellitus-related age-adjusted mortality rates (AAMRs) per 10,00,000 in Adults in the United States, 1999 to 2020. AAMRs are mapped to illustrate state-level variation in mortality burden. AAMR = age-adjusted mortality rate.

AAMR varied markedly among states, ranging from 10.93 in Nevada (95% CI: 9.48–12.38) to 37.6 in Nebraska (95% CI: 34.57–40.63). The AAMRs of states in the top 90th percentile, such as Nebraska, Ohio, California, Oklahoma, and Mississippi, were approximately twice as high as those of states in the bottom 10th percentile, including Delaware, Montana, Florida, Arizona, and Nevada (Fig. 4; Table S7, Supplemental Digital Content, https://links.lww.com/MD/Q856).

#### 3.5.2. Census region

**Figure 5.**
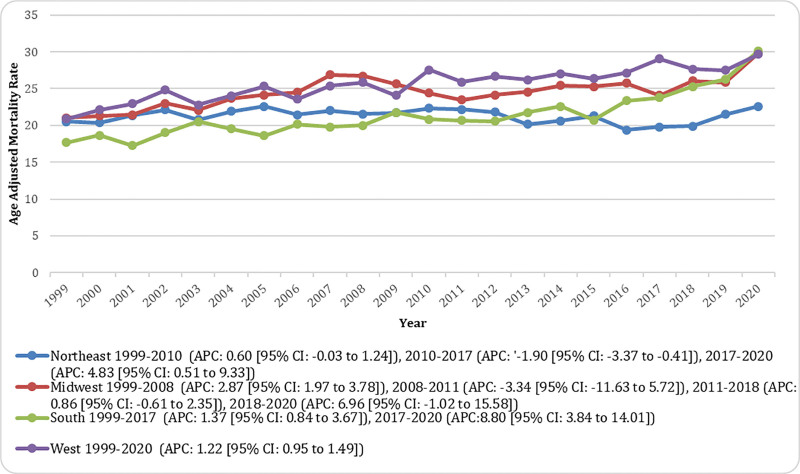
Census-stratified pancreatic cancer and diabetes mellitus-related age-adjusted mortality rates (AAMRs) per 10,00,000 in adults in the United States, 1999 to 2020. Trends are shown for Northeast, Midwest, South, and West, with Joinpoint-derived annual percent change (APC) and 95% confidence intervals shown for each trend segment. AAMR = age-adjusted mortality rate, APC = annual percent change, CI = confidence interval, NH = non-Hispanic.

Throughout the study period, mortality was highest in the Western region (overall AAMR: 25.92 [95% CI: 10.80–11.10]), followed by the Midwestern region (overall AAMR: 24.71 [95% CI: 24.30–25.11]), the Southern region (overall AAMR: 21.74 [95% CI: 21.44–22.03]), and the Northeastern region (overall AAMR: 21.25 [95% CI: 20.85–21.66]). The West showed a consistent upward trend, with AAMR increasing from 20.87 in 1999 to 29.70 in 2020 (APC: 1.22 [95% CI: 0.95–1.49]). In the Midwest, mortality rose from 21.04 in 1999 to 26.75 in 2008 (APC: 2.87 [95% CI: 1.97–3.78]), declined nonsignificantly to 23.47 in 2011 (APC: −3.34 [95% CI: −11.63 to 5.72]), then increased gradually to 26.04 in 2018 (APC: 0.86 [95% CI: −0.61 to 2.35]) and rose sharply to 29.81 in 2020 (APC: 6.96 [95% CI: −1.02 to 15.58]). The South demonstrated a steady rise from 17.67 in 1999 to 23.78 in 2017 (APC: 1.37 [95% CI: 0.96–1.78]), followed by a marked increase to 26.37 in 2020 (APC: 8.80 [95% CI: 3.84–14.01]). In the Northeast, rates increased slightly from 20.53 in 1999 to 22.35 in 2010 (APC: 0.60 [95% CI: −0.03 to 1.24]), declined significantly to 19.80 by 2017 (APC: −1.90 [95% CI: −3.37 to −0.41]), and then rose sharply to 22.59 in 2020 (APC: 4.83 [95% CI: 0.51–9.33]; Fig. 5; Table S8, Supplemental Digital Content, https://links.lww.com/MD/Q856).

#### 3.5.3. *Urban–rural*

**Figure 6.**
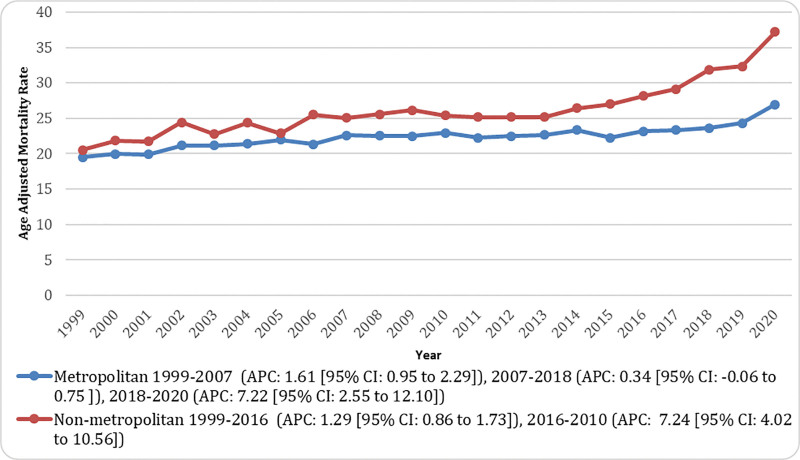
Urban and rural-stratified pancreatic cancer and diabetes mellitus-related age-adjusted mortality rates (AAMRs) per 10,00,000 in Adults in the United States, 1999 to 2020. Trends compare metropolitan (urban) vs nonmetropolitan (rural) areas based on the 2013 National Center for Health Statistics classification. All urban–rural comparisons are based on the 2013 NCHS Urban–Rural Classification for Counties and bridged-race denominators (1999–2020) for consistency across the full study period. AAMR = age-adjusted mortality rate, APC = annual percent change, CI = confidence interval.

Rural areas consistently exhibited higher AAMRs for combined deaths due to DM and PC compared with urban areas throughout the study period from 1999 to 2020. The overall AAMR in rural areas was 26.43 per 10,00,000 compared with 22.57 per 10,00,000 in urban areas. In rural regions, the AAMR increased significantly from 20.56 in 1999 to 28.15 in 2016 (APC: 1.29 [95% CI: 0.86–1.73]) and then rose significantly to 37.21 in 2020 (APC: 7.24 [95% CI: 4.02–10.56]). In urban areas, the AAMR increased significantly from 19.50 in 1999 to 22.61 in 2007 (APC: 1.61 [95% CI: 0.95–2.29]), followed by a non‑significant increase to 23.66 in 2018 (APC: 0.34 [95% CI: −0.06 to 0.75]) and then a significant rise to 26.67 in 2020 (APC: 7.22 [95% CI: 2.55–12.10]; Fig. 6; Tables S3 and S9, Supplemental Digital Content, https://links.lww.com/MD/Q856**).**

### 3.6. Sensitivity analysis

To account for potential misclassification of the underlying cause of death, a sensitivity analysis was conducted, restricting the dataset to cases in which PC was recorded as the underlying cause and DM as a contributing cause. Mortality continued to show a significant upward trend from 1999 to 2020 (AAPC: 1.50 [95% CI: 0.98–2.02]).

Stratified analyses showed consistent findings across subgroups. Both genders exhibited rising mortality, with a steeper increase among males (AAPC: 1.99 [95% CI: 1.22–2.76]) compared to females (AAPC: 0.94 [95% CI: 0.24–1.65]). Across racial groups, mortality increased among White (AAPC: 1.89 [95% CI: 1.30–2.49]) and Hispanic (AAPC: 0.51 [95% CI: −0.09 to 1.10]) populations, while declining trends were observed among Black (AAPC: −0.60 [95% CI: −1.02 to 0.18]) and Asian (AAPC: −0.81 [95% CI: −1.68 to 0.07]) populations.

Age-specific analysis revealed increases across all groups, most notably among those aged 45 to 54 years (AAPC: 2.28 [95% CI: 1.69–2.87]), followed by 55 to 64 (AAPC: 2.00 [95% CI: 0.53–3.50]) and 65 to 74 years (AAPC: 1.55 [95% CI: 1.05–2.04]). Mortality also rose modestly among individuals aged 75 to 84 (AAPC: 0.86 [95% CI: 0.58–1.14]) and 85 years and older (AAPC: 1.19 [95% CI: −0.00 to 2.39]).

Regionally, mortality remained stable in the Northeast (AAPC: −0.04 [95% CI: −0.87 to 0.80]) and South (AAPC: −0.09 [95% CI: −1.32 to 1.15]) but increased in the Midwest (AAPC: 1.39 [95% CI: −0.41 to 3.22]) and West (AAPC: 1.40 [95% CI: 1.24–1.55]). Both urban and rural areas showed upward trends, with rural regions exhibiting a greater rise (AAPC: 2.33 [95% CI: 1.60–3.08]) compared to urban regions (AAPC: 1.24 [95% CI: 0.75–1.73]).

These findings confirm that the increasing mortality trends persisted when restricted to cases with PC as the underlying cause of death, demonstrating the robustness of the main results.

## 4. Discussion

The study highlights significant patterns in the number of deaths in the US from 1999 to 2020 from DM and PC. By utilizing national death certificate data, we observed changing patterns over time, highlighting the ongoing public health burden of both diseases and their potential interconnection. First, there was an initial rise in the mortality rate from 1999 to 2004, followed by a gradual increase until 2018, which was then succeeded by a surge in 2020. This trend held for men; however, among women, a slight decline was observed from 2008 to 2017, followed by an increase. Second, in comparison to other racial and ethnic groupings, NH Black or African American people showed the highest AAMR, with a gradual increase over time. NH White experienced a notable rise in mortality rates, especially in the last few years. Third, mortality rates rose as age increased; individuals aged 85 and above had the highest AAMRs. The 45 to 54 and 75 to 84 age groups exhibited a steady rise in mortality throughout the study period, while the 55 to 64 and 85 + age groups initially showed an upward trend; both experienced periods of decline before a sharp increase in 2020. Fourth, AAMR in rural areas was consistently somewhat greater than in urban areas, although the difference remained modest. Fifth, there was considerable disparity in AAMRs of states, with the top 90th percentile exceeding the bottom 10th percentile by more than 3-fold. This notable geographic disparity can be a consequence of unequal access to healthcare, inadequate disease management, or population-level risk factors.

DM and PC have a complicated and 2-way relationship, with 1 causing the other.^[15]^ Two theories can explain the onset of DM in patients with PC. The first theory proposes that environmental factors, such as tobacco, may damage beta cells that produce insulin and promote tumor growth. The second theory suggests that hyperglycemia is caused by paraneoplastic syndrome, which results from tumor-derived substances that disrupt normal glucose metabolism.^[16,17]^ DM contributes to PC through several mechanisms, such as hyperglycemia, insulin resistance, hyperinsulinemia, IGFs, genetic predisposition, and increased oxidative stress.^[14]^ In addition to that, specific antidiabetic agents are known to increase the risk of PC.^[18]^ For example, individuals who start insulin therapy within the first year after being diagnosed with DM have a 71% higher chance of developing PC.^[19]^ Additionally, both diseases are associated with similar risk factors, such as obesity, insulin resistance, advanced age, genetic predisposition, smoking, low socioeconomic status, and African–American ethnicity.^[20,21]^

Over the past 2 decades, mortality from both DM and PC has risen drastically. This observation is consistent with findings from prior studies conducted by Zhu and co-workers^[22]^ and illustrates the growing burden that these conditions impose on public health. PC is one of the most lethal malignancies, with a 5-year survival rate of only 4% to 12%.^[23]^ Recent cohort data show that individuals with preexisting DM have significantly worse overall survival compared to nondiabetic individuals.^[24]^ This can be attributed to several factors. The increasing prevalence of lifestyle-related risk factors, such as tobacco use, physical inactivity, and poor eating habits, contributes to the development and progression of both diseases.^[25,26]^ While advancements in diagnostic tools and enhanced screening may contribute to improving detection rates, the underlying increase in risk factors remains the primary worry.^[26]^

Our findings build upon those of Tan et al in 2024,^[7]^ who recently analyzed nationwide PC mortality trends in the U.S using CDC WONDER data from 1999 to 2020 and reported no meaningful improvement in PC-specific mortality despite therapeutic advances. While that study provided essential insights into demographic and geographic disparities in PC mortality, it did not account for the co-existence of DM. This highly prevalent metabolic disorder shares biological and epidemiologic pathways with PC. By jointly analyzing deaths in which both PC and DM were contributing causes, our study extends their work by quantifying the additional mortality burden attributable to this comorbidity. This integrated approach highlights the compounded risk among individuals living with both conditions and underscores the necessity for coordinated oncologic and metabolic disease prevention strategies.

Our analysis revealed notable gender-based differences in mortality trends related to DM and PC. While AAMRs increased for both sexes, males consistently exhibited higher rates across all age groups. This observation aligns with previous studies by Nipp et al and Zhou et al,^[27,28]^ which also reported a higher incidence of both conditions in men. Several behavioral and biological factors may contribute to this disparity. Risk behaviors such as smoking and excessive alcohol consumption are more prevalent among men and are well-established contributors to the development of both DM and PC.^[28]^ Hormonal differences may also play a role. A systematic review by Gan et al^[29]^ suggests that estrogen may have a protective effect against PC via ER and GPER signaling pathways. In contrast, androgens lack this benefit and may potentially increase risk, though the exact mechanisms remain unclear. Additionally, a high body mass index (BMI) in early adulthood has been associated with an elevated risk of PC in men. In contrast, this association is not as evident in women, possibly due to sex-related differences in fat distribution, which also requires further investigation.^[30]^

Our study also revealed significant and persistent racial and ethnic disparities in mortality trends related to DM and PC, with NH Black individuals exhibiting the highest AAMRs. These disparities likely result from a combination of biological, socioeconomic, and healthcare system-related factors. In the case of DM, although prevalence is high among Black populations, they are more likely to remain undiagnosed.^[31]^ Even when diagnosed, disparities persist in access to essential technologies, such as continuous glucose monitoring (CGM), as well as in the prescription and use of recommended medications.^[32–34]^ Additionally, lifestyle-related risk factors such as obesity, physical inactivity, tobacco use, and poor diet are more prevalent in Black populations, contributing further to the burden of both DM and PC.^[35,36]^ Regarding PC, Black individuals not only experience higher incidence and mortality rates but are also more likely to present with late-stage disease and less likely to be evaluated for surgery or undergo curative resection, even after adjusting for disease stage, comorbidities, and socioeconomic status.^[37,38]^

Our findings showed a significant increase in mortality with advancing age, with individuals aged 85 years and older exhibiting the highest AAMRs. This trend aligns with a study by Tseng et al, which reported that individuals aged 70 and above bear the most significant burden of disease.^[39]^ The heightened vulnerability in older adults can be attributed to age-related metabolic dysfunction, chronic systemic inflammation, and long-term exposure to risk factors such as obesity, DM, and tobacco use.^[40]^ In addition, delayed diagnoses, limited treatment options, and a higher prevalence of comorbidities in this age group further contribute to poor outcomes.^[40]^ DM particularly when long-standing or newly diagnosed in older adults, also significantly increases the risk and worsens the prognosis of PC.^[41]^

Our study identified notable geographic disparities in mortality related to DM and PC. The Western region exhibited the highest overall AAMRs, with Nebraska having the most significant state-level mortality burden. Interestingly, while DM is a common cause of death in this region, the West reported the lowest PC mortality rates.^[9,42]^ This may reflect the fact that individuals with DM have approximately a 30% higher risk of death than those without the condition, regardless of cancer status.^[43]^ Significant urban–rural differences were also observed. Rural populations demonstrated higher mortality rates, driven mainly by a greater prevalence of key risk factors such as obesity, metabolic syndrome, tobacco use, alcohol abuse, and physical inactivity.^[44,45]^ These health challenges are compounded by limited access to healthcare resources and health education in rural settings. As a result, rural residents often experience poor DM management due to inadequate treatment adherence and lack of access to tools like continuous glucose monitoring, leading to worse glycemic control and greater risk of complications.^[46]^ Additionally, structural deficiencies in rural healthcare systems, exemplified by the closure of over 190 rural hospitals in the past 2 decades, have further reduced access to care.^[47]^ Long travel distances to medical facilities also pose a barrier to timely preventive care.^[48]^ In the context of PC, this lack of infrastructure contributes to delayed diagnoses, with most rural cases detected at later stages, resulting in poorer outcomes.^[49]^

## 5. Limitations

This retrospective analysis has some inherent limitations. First and foremost, it is based on death record information from the CDC WONDER database, which may contain errors due to misclassification or reporting mistakes.^[50]^ The lack of autopsy validation and the potential inconsistencies in death certificate documentation can undermine the accuracy of cause-of-death attribution.^[51,52]^ Moreover, the study does not differentiate between different types of DM, which may obscure significant variations in disease burden and outcomes. As an observational study, it is also constrained in its ability to establish causal relationships; future prospective cohort studies are needed to understand potential causal pathways better.

In addition, the analysis could not be standardized to the size of the DM population (e.g., compute mortality per person with DM) because CDC WONDER does not provide linked denominators by DM status. Consequently, we cannot fully distinguish whether rising AAMRs reflect worsening outcomes, an increase in the number of people living with DM, or both. Future work that links cause-of-death data with the National Health Interview Survey (NHIS) and the Behavioral Risk Factor Surveillance System (BRFSS) or other claims-based DM prevalence sources is needed to estimate DM-standardized mortality and directly assess case-fatality over time.

Finally, while the COVID-19 pandemic began globally in late 2019, its most acute impact on US healthcare systems was evident starting in March 2020; therefore, only the final year of the study period (2020) may have been affected by pandemic-related disruptions, which warrants further investigation in future analyses.

## 6. Conclusion

This comprehensive analysis of US mortality data from 1999 to 2020 highlights significant demographic and geographic disparities in deaths related to PC and DM. Older adults, males, NH Black populations, and rural residents consistently experienced the highest AAMR. Geographic hotspots, including states like Nebraska and regions such as the West, underscore persistent inequalities in access to care and chronic disease management. These findings call for integrated, equity-focused healthcare strategies including improved rural outreach, culturally tailored screening programs, and targeted preventive interventions for high-risk populations to reduce the burden of these increasingly lethal conditions.

## Acknowledgments

All authors would like to extend their sincere regards to the team of the Research Council of Pakistan (RCOP) for their guidance and mentorship.

## Author contributions

**Data curation**: Aqsa Shaikh, Abuzar Khan.

**Formal analysis**: Neelam Kumari, Muhammad Abdul Rafay.

**Project administration**: Asad Ali Ahmed Cheema.

**Supervision**: Asad Ali Ahmed Cheema.

**Writing – original draft**: Aqsa Shaikh.

**Writing – review & editing**: Asad Ali Ahmed Cheema, Neelam Kumari.

## Supplementary Material
