# Supplementary material for: Two decades of mortality trends in pancreatic cancer and diabetes mellitus: A retrospective cross-sectional study of the United States population (1999–2020)
Source: Medicine (Baltimore). 2026 May 12;104(49):e46372. doi: 10.1097/MD.0000000000046372 (PMC12689041; doi:10.1097/MD.0000000000046372)
Supplement: Supplementary file 1 [file medi-104-e46372-s001.docx]

|  | **Deaths** | | | | | | | | |
| --- | --- | --- | --- | --- | --- | --- | --- | --- | --- |
| **Year** | **Overall** | **Women** | **Men** | **NH White** | **NH Black or African American** | **NH Asian or Pacific Islander** | **NH American Indian or Alaska Native** | **Hispanic or Latino** | **Population** |
| 1999 | 1879 | 954 | 925 | 1524 | 294 | 55 | - | 127 | 95153686 |
| 2000 | 1962 | 1023 | 939 | 1611 | 305 | 37 | - | 108 | 96944389 |
| 2001 | 1982 | 981 | 1001 | 1649 | 289 | 35 | - | 120 | 99781854 |
| 2002 | 2166 | 1102 | 1064 | 1773 | 328 | 58 | - | 141 | 102217733 |
| 2003 | 2171 | 1076 | 1095 | 1784 | 321 | 56 | 10 | 150 | 104692428 |
| 2004 | 2255 | 1089 | 1166 | 1830 | 355 | 60 | 10 | 142 | 107138553 |
| 2005 | 2317 | 1127 | 1190 | 1876 | 355 | 74 | 12 | 180 | 109787199 |
| 2006 | 2363 | 1150 | 1213 | 1937 | 344 | 66 | 16 | 176 | 112380379 |
| 2007 | 2506 | 1202 | 1304 | 2062 | 358 | 76 | 10 | 179 | 114894084 |
| 2008 | 2568 | 1225 | 1343 | 2124 | 360 | 76 | - | 199 | 117395131 |
| 2009 | 2630 | 1240 | 1390 | 2130 | 404 | 87 | - | 215 | 119895863 |
| 2010 | 2711 | 1259 | 1452 | 2198 | 389 | 100 | 24 | 227 | 121757429 |
| 2011 | 2698 | 1301 | 1397 | 2191 | 379 | 111 | 17 | 217 | 124174484 |
| 2012 | 2807 | 1275 | 1532 | 2312 | 363 | 105 | 27 | 223 | 126000296 |
| 2013 | 2905 | 1338 | 1567 | 2363 | 417 | 102 | 23 | 250 | 127788037 |
| 2014 | 3064 | 1388 | 1676 | 2515 | 412 | 117 | 20 | 298 | 129779643 |
| 2015 | 3027 | 1337 | 1690 | 2493 | 397 | 117 | 20 | 289 | 131826832 |
| 2016 | 3244 | 1411 | 1833 | 2635 | 475 | 114 | 20 | 303 | 133494018 |
| 2017 | 3378 | 1405 | 1973 | 2782 | 449 | 115 | 32 | 353 | 135229289 |
| 2018 | 3568 | 1546 | 2022 | 2941 | 487 | 121 | 19 | 351 | 136335528 |
| 2019 | 3755 | 1604 | 2151 | 3056 | 535 | 134 | 30 | 342 | 137381702 |
| 2020 | 4257 | 1826 | 2431 | 3498 | 576 | 145 | 38 | 480 | 138429175 |
| **Total** | 60213 | 27859 | 32354 | 49284 | 8592 | 1961 | 376 | 5070 | 2622477732 |

**Supplemental Table 1.** Pancreatic Cancer and Diabetes Mellitus–related Deaths, Stratified by Sex and Race, in Adults in the United States, 1999 to 2020

NH, non-Hispanic.

**Supplemental Table 2.** Pancreatic Cancer and Diabetes Mellitus – related Mortality, Stratified by Place of Death in Adults in the United States, 1999 to 2020

|  | **Deaths** | | | |
| --- | --- | --- | --- | --- |
| **Year** | **Medical Facility** | **Decedent's home** | **Hospices** | **Nursing home/long term care** |
| 1999 | 666 | 777 | - | 335 |
| 2000 | 658 | 828 | - | 374 |
| 2001 | 663 | 828 | - | 373 |
| 2002 | 713 | 907 | - | 420 |
| 2003 | 724 | 945 | - | 384 |
| 2004 | 681 | 960 | 18 | 444 |
| 2005 | 686 | 1025 | 42 | 422 |
| 2006 | 682 | 1042 | 76 | 426 |
| 2007 | 755 | 1060 | 113 | 446 |
| 2008 | 692 | 1134 | 133 | 439 |
| 2009 | 699 | 1141 | 165 | 418 |
| 2010 | 735 | 1199 | 171 | 424 |
| 2011 | 692 | 1183 | 204 | 442 |
| 2012 | 650 | 1300 | 276 | 418 |
| 2013 | 716 | 1350 | 245 | 445 |
| 2014 | 694 | 1481 | 314 | 428 |
| 2015 | 684 | 1453 | 331 | 444 |
| 2016 | 721 | 1612 | 380 | 406 |
| 2017 | 770 | 1637 | 391 | 456 |
| 2018 | 842 | 1713 | 425 | 443 |
| 2019 | 813 | 1856 | 491 | 451 |
| 2020 | 813 | 2453 | 404 | 381 |
| **Total** | 15749 | 27884 | 4179 | 9219 |

**Supplemental Table 3.** Annual percent change (APC) of Pancreatic Cancer and Diabetes Mellitus –related Age-Adjusted Mortality Rates per 1,000,000 in Adults in the United States, 1999 to 2020

| Year Interval | APC (95% CI) |
| --- | --- |
| Overall | - |
| 1999-2004 | 2.2420* (0.8377 - 3.6659) |
| 2004-2018 | 0.6744* (0.3955 - 0.954) |
| 2018-2020 | 8.0059* (3.3539 - 12.8673) |
| Men |  |
| 1999-2018 | 1.3148* (1.0613 - 1.569) |
| 2018-2020 | 7.7367* (1.0187 - 14.9015) |
| Women |  |
| 1999-2008 | 1.2313* (0.4742 - 1.9942) |
| 2008-2017 | -0.5085 (-1.3202 - 0.3099) |
| 2017-2020 | 5.6044* (2.1801 - 9.1435) |
| NH White | - |
| 1999-2018 | 1.2222* (1.0141 - 1.4308) |
| 2018-2020 | 7.5967* (1.826 - 13.6943) |
| NH Black or African American | - |
| 1999-2020 | -0.4760* (-0.9129 - -0.0373) |
| NH American Indian or Alaska Native | - |
| 1999-2019 | - |
| Hispanic or Latino | - |
| 1999-2020 | 0.8246* (0.1922 - 1.461) |
| NH Asian or Pacific Islander | - |
| 1999-2020 | -0.6704 (-1.5415 - 0.2083) |
| Nonmetropolitan areas | - |
| 1999-2012 | 1.2907* (0.8571 - 1.7262) |
| 2012-2019 | 7.2399* (4.0188 - 10.5608) |
| Metropolitan area | - |
| 1999-2007 | 1.6141* (0.9451 - 2.2877) |
| 2007-2018 | 0.3415 (-0.0625 - 0.7471) |
| 2018-2020 | 7.2209* (2.5533 - 12.1009) |

APC = annual percent change; NH = non-Hispanic.

**Supplemental Table 4.** Overall and Sex‐Stratified Pancreatic Cancer and Diabetes Mellitus –related Age-Adjusted Mortality Rates per 1,000,000 in Adults in the United States, 1999 to 2020

|  | **Age-Adjusted Rate (95% CI)** | | |
| --- | --- | --- | --- |
| **Year** | **Men** | **Women** | **Overall** |
| 1999 | 23.51 (21.97 - 25.05) | 16.96 (15.88 - 18.04) | 19.72 (20.61 - 18.83) |
| 2000 | 23.46 (21.94 - 24.98) | 17.97 (16.87 - 19.08) | 20.36 (21.26 - 19.45) |
| 2001 | 24.57 (23.02 - 26.11) | 16.98 (15.91 - 18.05) | 20.24 (21.13 - 19.35) |
| 2002 | 25.60 (24.04 - 27.16) | 18.89 (17.77 - 20.01) | 21.76 (22.67 - 20.84) |
| 2003 | 25.70 (24.15 - 27.24) | 18.22 (17.13 - 19.32) | 21.43 (22.34 - 20.53) |
| 2004 | 26.69 (25.14 - 28.25) | 18.12 (17.04 - 19.20) | 21.92 (22.83 - 21.02) |
| 2005 | 26.90 (25.35 - 28.46) | 18.63 (17.54 - 19.72) | 22.13 (23.04 - 21.23) |
| 2006 | 26.66 (25.14 - 28.19) | 18.66 (17.58 - 19.75) | 22.14 (23.03 - 21.24) |
| 2007 | 27.85 (26.31 - 29.38) | 19.29 (18.19 - 20.38) | 23.04 (23.94 - 22.13) |
| 2008 | 27.89 (26.37 - 29.41) | 19.15 (18.07 - 20.23) | 23.07 (23.96 - 22.17) |
| 2009 | 28.01 (26.51 - 29.50) | 19.10 (18.03 - 20.17) | 23.13 (24.02 - 22.24) |
| 2010 | 28.75 (27.24 - 30.26) | 19.09 (18.02 - 20.15) | 23.41 (24.29 - 22.52) |
| 2011 | 26.89 (25.46 - 28.33) | 19.40 (18.34 - 20.47) | 22.71 (23.58 - 21.85) |
| 2012 | 28.44 (26.99 - 29.89) | 18.60 (17.57 - 19.63) | 22.94 (23.8 - 22.08) |
| 2013 | 28.27 (26.85 - 29.70) | 18.84 (17.82 - 19.86) | 23.09 (23.94 - 22.24) |
| 2014 | 29.42 (27.98 - 30.86) | 19.31 (18.28 - 20.34) | 23.84 (24.7 - 22.99) |
| 2015 | 28.87 (27.47 - 30.28) | 18.27 (17.28 - 19.27) | 23.06 (23.9 - 22.23) |
| 2016 | 30.50 (29.07 - 31.92) | 18.83 (17.84 - 19.83) | 24.02 (24.86 - 23.18) |
| 2017 | 31.60 (30.17 - 33.02) | 18.31 (17.34 - 19.28) | 24.27 (25.1 - 23.44) |
| 2018 | 31.54 (30.14 - 32.94) | 19.59 (18.60 - 20.58) | 25.01 (25.84 - 24.18) |
| 2019 | 32.46 (31.06 - 33.86) | 19.95 (18.96 - 20.94) | 25.63 (26.46 - 24.8) |
| 2020 | 36.60 (35.12 - 38.08) | 22.11 (21.08 - 23.13) | 28.61 (29.48 - 27.74) |

**Supplemental Table 5.** Pancreatic Cancer and Diabetes Mellitus – related Age-Adjusted Mortality Rates per 1,000,000, Stratified by Race in Adults in the United States, 1999 to 2020

|  | **Age-Adjusted Rate (95% CI)** | | | | |
| --- | --- | --- | --- | --- | --- |
| **Year** | **NH White** | **NH Black or African American** | **NH American Indian or Alaska Native** | **Hispanic or Latino** | **NH Asian or Pacific Islander** |
| 1999 | 18.01 (17.11 - 18.92) | 36.15 (31.98 - 40.32) | - | 27.39 (22.45 - 32.33) | 25.03 (18.64 - 32.91) |
| 2000 | 18.86 (17.94 - 19.78) | 37.35 (33.12 - 41.58) | - | 22.55 (18.14 - 26.95) | 15.60 (10.81 - 21.80) |
| 2001 | 19.07 (18.15 - 19.99) | 34.72 (30.68 - 38.76) | - | 22.94 (18.71 - 27.17) | 14.39 (9.91 - 20.21) |
| 2002 | 20.21 (19.27 - 21.15) | 37.99 (33.83 - 42.16) | - | 26.88 (22.31 - 31.45) | 21.57 (16.20 - 28.14) |
| 2003 | 20.06 (19.13 - 21.00) | 36.18 (32.16 - 40.20) | Unreliable (8.23 - 34.18) | 25.39 (21.20 - 29.58) | 20.23 (15.15 - 26.46) |
| 2004 | 20.29 (19.36 - 21.22) | 38.85 (34.74 - 42.96) | Unreliable (6.44 - 29.4) | 23.50 (19.51 - 27.49) | 19.63 (14.87 - 25.44) |
| 2005 | 20.52 (19.59 - 21.45) | 39.25 (35.10 - 43.41) | Unreliable (9.41 - 33.73) | 27.30 (23.18 - 31.43) | 23.05 (17.93 - 29.17) |
| 2006 | 20.79 (19.86 - 21.72) | 35.61 (31.76 - 39.46) | Unreliable (15.92 - 46.9) | 25.66 (21.75 - 29.57) | 19.26 (14.77 - 24.69) |
| 2007 | 21.76 (20.82 - 22.71) | 36.52 (32.65 - 40.39) | Unreliable (7.48 - 31.03) | 25.13 (21.33 - 28.92) | 21.95 (17.17 - 27.64) |
| 2008 | 21.97 (21.03 - 22.91) | 35.83 (32.04 - 39.62) | - | 26.00 (22.26 - 29.73) | 21.06 (16.51 - 26.48) |
| 2009 | 21.66 (20.74 - 22.59) | 39.26 (35.33 - 43.19) | - | 26.81 (23.10 - 30.51) | 21.56 (17.15 - 26.77) |
| 2010 | 21.96 (21.04 - 22.89) | 36.76 (33.00 - 40.51) | 31.36 (19.41 - 47.94) | 27.17 (23.51 - 30.83) | 23.26 (18.57 - 27.95) |
| 2011 | 21.46 (20.56 - 22.37) | 34.16 (30.62 - 37.71) | Unreliable (8.77 - 26.91) | 24.88 (21.47 - 28.29) | 25.30 (20.49 - 30.10) |
| 2012 | 22.07 (21.16 - 22.98) | 31.07 (27.77 - 34.37) | 27.53 (17.64 - 40.96) | 24.28 (20.99 - 27.56) | 22.26 (17.91 - 26.61) |
| 2013 | 21.98 (21.09 - 22.87) | 35.01 (31.54 - 38.48) | 24.09 (14.71 - 37.2) | 24.78 (21.61 - 27.94) | 20.20 (16.20 - 24.20) |
| 2014 | 22.98 (22.08 - 23.89) | 32.73 (29.47 - 36.00) | 19.64 (11.64 - 31.05) | 28.60 (25.25 - 31.95) | 21.10 (17.19 - 25.01) |
| 2015 | 22.39 (21.50 - 23.28) | 30.80 (27.67 - 33.92) | 19.36 (11.48 - 30.6) | 25.19 (22.18 - 28.20) | 19.61 (15.97 - 23.24) |
| 2016 | 23.09 (22.19 - 23.98) | 35.39 (32.10 - 38.68) | 16.2 (9.6 - 25.6) | 26.06 (23.02 - 29.10) | 18.71 (15.21 - 22.22) |
| 2017 | 23.78 (22.88 - 24.67) | 31.78 (28.75 - 34.82) | 24.4 (16.21 - 35.27) | 28.57 (25.49 - 31.65) | 17.09 (13.91 - 20.27) |
| 2018 | 24.49 (23.59 - 25.38) | 33.69 (30.60 - 36.77) | Unreliable (9.56 - 26.29) | 27.41 (24.44 - 30.37) | 17.81 (14.59 - 21.03) |
| 2019 | 24.83 (23.94 - 25.72) | 35.53 (32.43 - 38.64) | 22.15 (14.6 - 32.23) | 25.13 (22.38 - 27.88) | 18.39 (15.23 - 21.56) |
|  | 28.11 (27.17 - 29.05) | 36.98 (33.88 - 40.09) | 27.57 (19.2 - 38.34) | 33.92 (30.79 - 37.05) | 19.28 (16.10 - 22.47) |

    NH = non-Hispanic.

**Supplemental Table 6.** Pancreatic Cancer and Diabetes Mellitus – related Crude Mortality Rate per 1,000,000, Stratified by Age Group in Adults in the United States, 1999 to 2020

|  | Age-Adjusted Rate (95% CI) | | | | |
| --- | --- | --- | --- | --- | --- |
| Year | **45-54 Year** | **55-64 Year** | **65-74 Year** | **75-84 Year** | **85+ Year** |
| 1999 | 2.38 (1.91–2.93) | 10.85 (9.53–12.17) | 31.27 (28.72–33.83) | 57.10 (52.86–61.33) | 62.59 (54.98–70.20) |
| 2000 | 2.10 (1.66–2.61) | 10.67 (9.37–11.97) | 32.52 (29.91–35.12) | 61.40 (57.03–65.77) | 62.98 (55.42–70.53) |
| 2001 | 1.83 (1.43–2.30) | 10.20 (8.95–11.45) | 33.62 (30.97–36.27) | 59.55 (55.29–63.82) | 66.32 (58.63–74.01) |
| 2002 | 2.13 (1.70–2.63) | 11.38 (10.10–12.66) | 35.84 (33.10–38.57) | 62.99 (58.63–67.34) | 71.87 (63.92–79.82) |
| 2003 | 2.13 (1.71–2.63) | 11.89 (10.61–13.17) | 34.76 (32.07–37.44) | 62.42 (58.11–66.73) | 67.84 (60.20–75.48) |
| 2004 | 2.31 (1.87–2.82) | 11.91 (10.66–13.16) | 34.71 (32.04–37.39) | 63.66 (59.33–68.00) | 73.69 (65.80–81.58) |
| 2005 | 2.59 (2.10–3.07) | 11.42 (10.23–12.62) | 34.80 (32.13–37.46) | 64.32 (59.97–68.67) | 76.49 (68.58–84.40) |
| 2006 | 2.29 (1.86–2.78) | 12.56 (11.33–13.79) | 34.89 (32.25–37.53) | 63.38 (59.07–67.69) | 74.60 (66.93–82.27) |
| 2007 | 2.28 (1.83–2.72) | 12.83 (11.61–14.05) | 34.93 (32.32–37.54) | 69.84 (65.31–74.37) | 75.21 (67.63–82.78) |
| 2008 | 2.41 (1.95–2.86) | 12.79 (11.59–13.99) | 36.19 (33.58–38.79) | 65.39 (61.00–69.77) | 82.18 (74.39–89.98) |
| 2009 | 2.90 (2.40–3.40) | 13.27 (12.07–14.47) | 35.46 (32.93–38.00) | 66.35 (61.92–70.77) | 76.95 (69.53–84.37) |
| 2010 | 2.76 (2.27–3.24) | 13.92 (12.71–15.14) | 34.59 (32.11–37.06) | 66.69 (62.26–71.12) | 83.19 (75.56–90.82) |
| 2011 | 2.44 (1.98–2.90) | 12.66 (11.53–13.79) | 35.94 (33.46–38.42) | 65.12 (60.76–69.48) | 76.87 (69.69–84.04) |
| 2012 | 2.89 (2.39–3.39) | 13.63 (12.47–14.80) | 34.31 (31.97–36.66) | 65.62 (61.27–69.98) | 77.96 (70.83–85.10) |
| 2013 | 2.56 (2.09–3.03) | 13.43 (12.28–14.58) | 35.53 (33.21–37.86) | 64.70 (60.40–69.00) | 82.61 (75.36–89.85) |
| 2014 | 2.65 (2.16–3.13) | 13.70 (12.55–14.84) | 36.67 (34.36–38.98) | 70.45 (66.01–74.90) | 75.95 (69.07–82.83) |
| 2015 | 2.94 (2.43–3.45) | 11.96 (10.90–13.02) | 37.24 (34.96–39.52) | 69.09 (64.73–73.46) | 67.28 (60.87–73.69) |
| 2016 | 2.71 (2.22–3.20) | 13.19 (12.09–14.30) | 39.05 (36.76–41.34) | 70.47 (66.11–74.83) | 72.10 (65.51–78.69) |
| 2017 | 3.21 (2.67–3.75) | 13.55 (12.44–14.66) | 41.00 (38.70–43.30) | 67.66 (63.45–71.86) | 71.27 (64.76–77.77) |
| 2018 | 3.17 (2.63–3.71) | 14.34 (13.19–15.48) | 41.42 (39.14–43.70) | 70.29 (66.10–74.47) | 74.11 (67.51–80.70) |
| 2019 | 3.30 (2.75–3.86) | 15.36 (14.18–16.54) | 42.69 (40.41–44.97) | 69.44 (65.36–73.53) | 77.97 (71.24–84.71) |
| 2020 | 3.72 (3.12–4.31) | 16.67 (15.44–17.90) | 44.89 (42.58–47.19) | 81.94 (77.56–86.31) | 88.76 (81.60–95.92) |

**Supplemental Table 7.** Pancreatic Cancer and Diabetes Mellitus – related Age-Adjusted Mortality Rates per 1,000,000 Stratified by States in Adults in the United States, 1999 to 2020

| **State** | **Age-Adjusted Rate (95% CI)** |
| --- | --- |
| Alabama | 17.33 (16.05 - 18.6) |
| Alaska | 19.48 (15.03 - 24.83) |
| Arizona | 12.96 (12 - 13.91) |
| Arkansas | 19.7 (18 - 21.41) |
| California | 30.96 (30.31 - 31.62) |
| Colorado | 26.2 (24.51 - 27.89) |
| Connecticut | 21.22 (19.63 - 22.81) |
| Delaware | 15.71 (12.98 - 18.44) |
| District of Columbia | 25.46 (20.81 - 30.11) |
| Florida | 14.29 (13.77 - 14.81) |
| Georgia | 17.31 (16.3 - 18.31) |
| Hawaii | 22.66 (20.01 - 25.31) |
| Idaho | 26.36 (23.48 - 29.24) |
| Illinois | 20.05 (19.18 - 20.91) |
| Indiana | 24.74 (23.4 - 26.07) |
| Iowa | 23.74 (21.96 - 25.52) |
| Kansas | 18.63 (16.9 - 20.36) |
| Kentucky | 29.56 (27.8 - 31.32) |
| Louisiana | 19.74 (18.28 - 21.19) |
| Maine | 20.81 (18.36 - 23.27) |
| Maryland | 26.4 (24.9 - 27.9) |
| Massachusetts | 17.52 (16.45 - 18.6) |
| Michigan | 23.89 (22.86 - 24.92) |
| Minnesota | 29.35 (27.75 - 30.95) |
| Mississippi | 29.88 (27.7 - 32.07) |
| Missouri | 19.42 (18.23 - 20.61) |
| Montana | 15.11 (12.59 - 17.63) |
| Nebraska | 37.6 (34.57 - 40.63) |
| Nevada | 10.93 (9.48 - 12.38) |
| New Hampshire | 24.73 (21.85 - 27.61) |
| New Jersey | 22.27 (21.21 - 23.33) |
| New Mexico | 20.33 (18.16 - 22.5) |
| New York | 18.26 (17.62 - 18.91) |
| North Carolina | 23.75 (22.66 - 24.84) |
| North Dakota | 25.65 (21.61 - 29.7) |
| Ohio | 31.59 (30.5 - 32.67) |
| Oklahoma | 30.4 (28.49 - 32.32) |
| Oregon | 29.03 (27.21 - 30.84) |
| Pennsylvania | 25.88 (24.98 - 26.77) |
| Rhode Island | 22.61 (19.63 - 25.58) |
| South Carolina | 20.61 (19.19 - 22.03) |
| South Dakota | 25.95 (22.3 - 29.6) |
| Tennessee | 22.53 (21.26 - 23.81) |
| Texas | 28.91 (28.09 - 29.72) |
| Utah | 17.91 (15.83 - 19.99) |
| Vermont | 25.04 (20.95 - 29.13) |
| Virginia | 18.41 (17.34 - 19.48) |
| Washington | 24.02 (22.7 - 25.34) |
| West Virginia | 25.43 (23.13 - 27.73) |
| Wisconsin | 21.95 (20.65 - 23.24) |
| Wyoming | 23.88 (19.35 - 28.42) |

**Supplemental Table 8.** Pancreatic Cancer and Diabetes Mellitus – related Age-Adjusted Mortality Rates per 1,000,000, Stratified by Census Region in Adults in the United States, 1999 to 2020

|  | Age-Adjusted Rate (95% CI) | | | |
| --- | --- | --- | --- | --- |
| Year | **Northeast** | **Midwest** | **South** | **West** |
| 1999 | 20.53 (18.55 - 22.52) | 21.04 (19.15 - 22.94) | 17.67 (16.25 - 19.09) | 20.87 (18.82 - 22.93) |
| 2000 | 20.38 (18.42 - 22.35) | 21.3 (19.4 - 23.2) | 18.67 (17.22 - 20.12) | 22.15 (20.05 - 24.26) |
| 2001 | 21.37 (19.37 - 23.38) | 21.46 (19.57 - 23.36) | 17.27 (15.89 - 18.66) | 22.95 (20.84 - 25.07) |
| 2002 | 22.11 (20.09 - 24.14) | 23 (21.05 - 24.95) | 19.01 (17.57 - 20.45) | 24.81 (22.64 - 26.99) |
| 2003 | 20.79 (18.83 - 22.75) | 22.08 (20.19 - 23.98) | 20.54 (19.06 - 22.02) | 22.78 (20.72 - 24.84) |
| 2004 | 21.93 (19.93 - 23.93) | 23.66 (21.7 - 25.62) | 19.54 (18.11 - 20.97) | 24.02 (21.92 - 26.11) |
| 2005 | 22.62 (20.58 - 24.65) | 24.17 (22.21 - 26.14) | 18.61 (17.22 - 20) | 25.35 (23.24 - 27.47) |
| 2006 | 21.45 (19.49 - 23.42) | 24.49 (22.53 - 26.45) | 20.17 (18.74 - 21.59) | 23.58 (21.56 - 25.61) |
| 2007 | 22.02 (20.04 - 24.01) | 26.87 (24.83 - 28.92) | 19.81 (18.42 - 21.21) | 25.4 (23.31 - 27.48) |
| 2008 | 21.54 (19.59 - 23.5) | 26.75 (24.73 - 28.77) | 20 (18.62 - 21.38) | 25.87 (23.79 - 27.94) |
| 2009 | 21.74 (19.78 - 23.69) | 25.63 (23.66 - 27.6) | 21.75 (20.33 - 23.18) | 24.09 (22.12 - 26.07) |
| 2010 | 22.35 (20.39 - 24.32) | 24.39 (22.48 - 26.31) | 20.84 (19.46 - 22.22) | 27.57 (25.48 - 29.66) |
| 2011 | 22.16 (20.21 - 24.11) | 23.47 (21.62 - 25.32) | 20.67 (19.31 - 22.03) | 25.88 (23.88 - 27.89) |
| 2012 | 21.84 (19.92 - 23.75) | 24.15 (22.28 - 26.01) | 20.56 (19.23 - 21.89) | 26.67 (24.67 - 28.66) |
| 2013 | 20.17 (18.36 - 21.99) | 24.57 (22.7 - 26.44) | 21.79 (20.43 - 23.14) | 26.23 (24.29 - 28.17) |
| 2014 | 20.61 (18.78 - 22.44) | 25.46 (23.58 - 27.34) | 22.6 (21.24 - 23.95) | 27.06 (25.1 - 29.02) |
| 2015 | 21.29 (19.42 - 23.16) | 25.27 (23.4 - 27.14) | 20.72 (19.44 - 22.01) | 26.39 (24.49 - 28.28) |
| 2016 | 19.37 (17.62 - 21.12) | 25.74 (23.86 - 27.62) | 23.39 (22.05 - 24.73) | 27.14 (25.25 - 29.04) |
| 2017 | 19.8 (18.07 - 21.53) | 24.09 (22.31 - 25.87) | 23.78 (22.45 - 25.12) | 29.06 (27.13 - 31) |
| 2018 | 19.92 (18.18 - 21.66) | 26.04 (24.21 - 27.87) | 25.28 (23.93 - 26.63) | 27.68 (25.82 - 29.54) |
| 2019 | 21.51 (19.73 - 23.29) | 25.85 (24.05 - 27.66) | 26.27 (24.91 - 27.63) | 27.53 (25.71 - 29.34) |
| 2020 | 22.59 (20.78 - 24.4) | 29.81 (27.89 - 31.74) | 30.13 (28.68 - 31.58) | 29.7 (27.83 - 31.57) |
| Total | 21.25 (20.85 - 21.66) | 24.71 (24.3 - 25.11) | 21.74 (21.44 - 22.03) | 25.92 (25.49 - 26.34) |

**Supplemental Table 9.** Pancreatic Cancer and Diabetes Mellitus – related Age-Adjusted Mortality Rates per 1,000,000, Stratified by Urban-Rural Classification in Adults in the United States, 1999 to 2020

|  | **Age-Adjusted Rate (95% CI)** | |
| --- | --- | --- |
| **Year** | **Metropolitan** | **Nonmetropolitan** |
| 1999 | 19.5 (18.52 - 20.48) | 20.56 (18.47 - 22.66) |
| 2000 | 19.99 (19 - 20.98) | 21.88 (19.73 - 24.03) |
| 2001 | 19.89 (18.91 - 20.87) | 21.74 (19.6 - 23.87) |
| 2002 | 21.18 (20.18 - 22.19) | 24.42 (22.17 - 26.67) |
| 2003 | 21.17 (20.17 - 22.16) | 22.79 (20.62 - 24.95) |
| 2004 | 21.39 (20.4 - 22.38) | 24.37 (22.14 - 26.59) |
| 2005 | 21.98 (20.98 - 22.98) | 22.87 (20.72 - 25.01) |
| 2006 | 21.35 (20.37 - 22.32) | 25.54 (23.29 - 27.78) |
| 2007 | 22.61 (21.62 - 23.6) | 25.07 (22.86 - 27.28) |
| 2008 | 22.52 (21.54 - 23.5) | 25.56 (23.35 - 27.77) |
| 2009 | 22.48 (21.51 - 23.45) | 26.18 (23.95 - 28.42) |
| 2010 | 22.97 (21.99 - 23.94) | 25.42 (23.23 - 27.6) |
| 2011 | 22.27 (21.32 - 23.22) | 25.16 (23.01 - 27.31) |
| 2012 | 22.49 (21.56 - 23.43) | 25.17 (23.03 - 27.31) |
| 2013 | 22.66 (21.74 - 23.59) | 25.18 (23.06 - 27.29) |
| 2014 | 23.33 (22.4 - 24.26) | 26.44 (24.28 - 28.6) |
| 2015 | 22.26 (21.36 - 23.16) | 27.03 (24.86 - 29.2) |
| 2016 | 23.18 (22.27 - 24.08) | 28.15 (25.96 - 30.34) |
| 2017 | 23.34 (22.45 - 24.24) | 29.14 (26.93 - 31.36) |
| 2018 | 23.66 (22.77 - 24.55) | 31.87 (29.59 - 34.15) |
| 2019 | 24.31 (23.43 - 25.2) | 32.32 (30.03 - 34.6) |
| 2020 | 26.97 (26.05 - 27.9) | 37.21 (34.76 - 39.67) |

**Supplemental Figure 1.** Two Decades of Mortality Trends in Pancreatic Cancer and Diabetes Mellitus: A Retrospective Cross-Sectional Study of the United States Population (1999–2020)

**
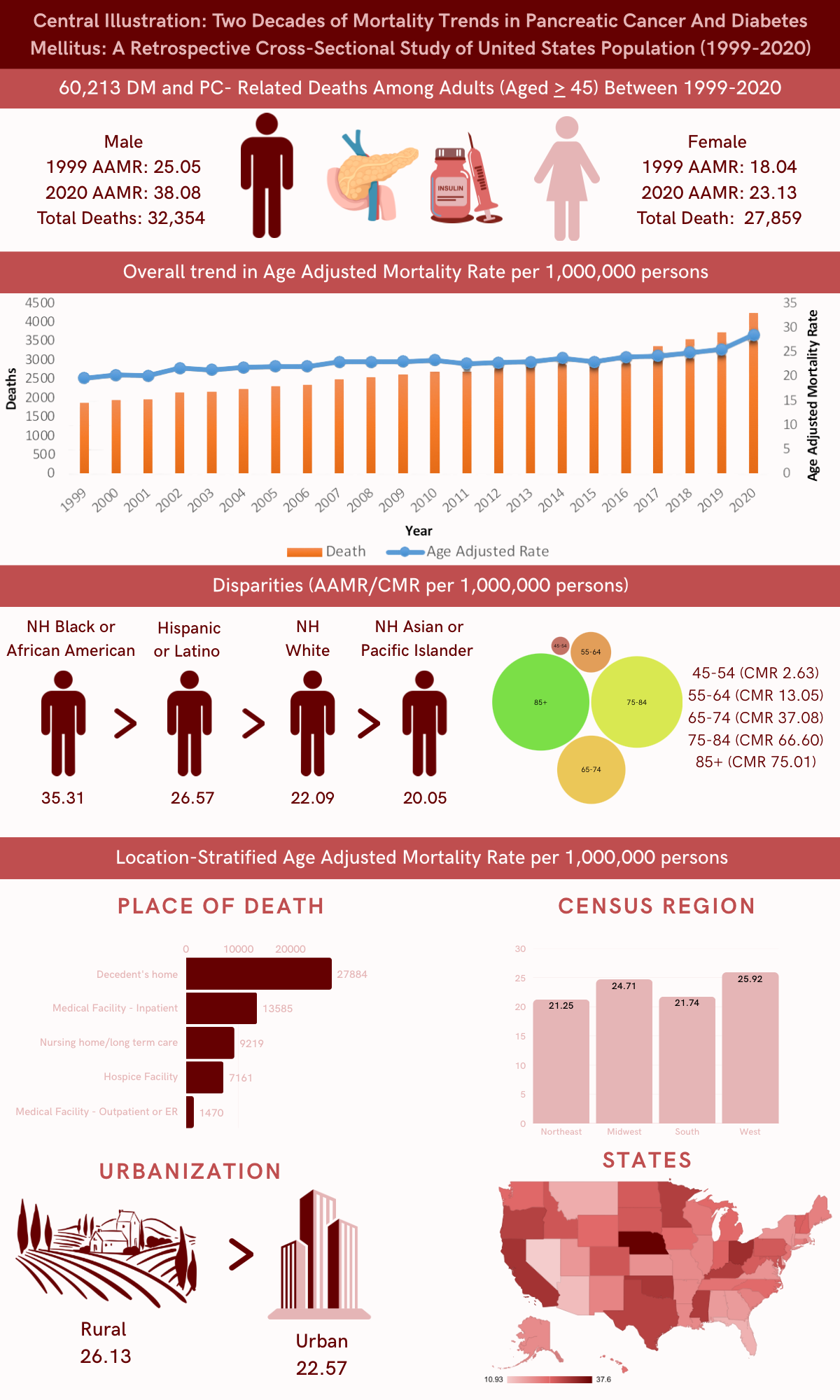
**
